# Temporal Discrimination Thresholds and Proprioceptive Performance: Impact of Age and Nerve Conduction

**DOI:** 10.3389/fnins.2019.01241

**Published:** 2019-11-19

**Authors:** Thorsten M. Odorfer, Teresa Wind, Daniel Zeller

**Affiliations:** Department of Neurology, University of Würzburg, Würzburg, Germany

**Keywords:** pointing task, position estimation, aging, kinesthesia, somatosensory temporal discrimination, TDMT, temporal discrimination threshold

## Abstract

**Background:**

Increasing attention is payed to the contribution of somatosensory processing in motor control. In particular, temporal somatosensory discrimination has been found to be altered differentially in common movement disorders. To date, there have only been speculations as to how impaired temporal discrimination and clinical motor signs may relate to each other. Prior to disentangling this relationship, potential confounders of temporal discrimination, in particular age and peripheral nerve conduction, should be assessed, and a quantifiable measure of proprioceptive performance should be established.

**Objective:**

To assess the influence of age and polyneuropathy (PNP) on somatosensory temporal discrimination threshold (STDT), temporal discrimination movement threshold (TDMT), and behavioral measures of proprioception of upper and lower limbs.

**Methods:**

STDT and TDMT were assessed in 79 subjects (54 healthy, 25 with PNP; age 30–79 years). STDT was tested with surface electrodes over the thenar or dorsal foot region. TDMT was probed with needle electrodes in flexor carpi radialis (FCR) and tibialis anterior (TA) muscle. Goniometer-based devices were used to assess limb proprioception during (i) active pointing to LED markers, (ii) active movements in response to variable visual cues, and (iii) estimation of limb position following passive movements. Pointing (or estimation) error was taken as a measure of proprioceptive performance.

**Results:**

In healthy subjects, higher age was associated with higher STDT and TDMT at upper and lower extremities, while age did not correlate with proprioceptive performance. Patients with PNP showed higher STDT and TDMT values and decreased proprioceptive performance in active pointing tasks compared to matched healthy subjects. As an additional finding, there was a significant correlation between performance in active pointing tasks and temporal discrimination thresholds.

**Conclusion:**

Given their notable impact on measures of temporal discrimination, age and peripheral nerve conduction need to be accounted for if STDT and TDMT are applied in patients with movement disorders. As a side observation, the correlation between measures of proprioception and temporal discrimination may prompt further studies on the presumptive link between these two domains.

## Background

Temporal aspects of somatosensory processing have drawn increasing interest as potential markers in the differential diagnostic workup of movement disorders. In particular, STDT and TDMT have been shown to be differentially involved. STDT is a neurophysiological paradigm testing the shortest ISI at which a subject can perceive successive electrical stimuli applied to the skin as separate. Higher STDTs have been consistently described in several types of dystonia (Tinazzi et al., 1999, 2004; Bara-Jimenez et al., 2000; Aglioti et al., 2003; Fiorio et al., 2003, 2008) as well as in PD (Artieda et al., 1992; Conte et al., 2010, 2016) and multiple system atrophy (Rocchi et al., 2013). TDMT is defined as the shortest interval at which a subject perceives two externally induced passive movements as separate (Tinazzi et al., 2005). Compared to healthy controls, TDMT has been shown to be increased in PD patients (Fiorio et al., 2007) and patients with essential or functional tremor (Tinazzi et al., 2013a, 2014), whereas it was found normal in patients with writer’s cramp (Tinazzi et al., 2006) and dystonia with tremor (Tinazzi et al., 2013a).

To date, there have only been speculations as to how an impairment of temporal discrimination performance and clinical motor signs in these movement disorders may relate to each other (Riemann and Lephart, 2002a, b; Lee et al., 2016), warranting the need for further research. However, prior to disentangling this relationship on the CNS level, we consider it reasonable to examine how temporal discrimination is influenced by age and peripheral nerve conduction – factors which are likely to confound analyses in groups of movement disorder patients with high inter-group heterogeneity. Moreover, a quantifiable measure of proprioceptive performance should be established in order to assess potential associations between temporal discrimination and kinesthesia later on.

To this end, we assessed STDT and TDMT of upper and lower extremities in healthy subjects of different age and in patients with PNP, combined with a set of three proprioception tasks of the corresponding limbs. We hypothesized that higher age and PNP are associated with (i) increased discrimination thresholds and (ii) decreased proprioceptive performance.

## Materials and Methods

The study conformed to the principles of the Declaration of Helsinki. It was approved by the Ethics committee of the Medical Faculty at the University of Würzburg.

### Subjects

A total of 54 volunteers without a history of neurological or psychiatric disease and without clinical symptoms or signs of such disease were included. Additional exclusion criteria were a medical history of diabetes or coagulation disorders, and ongoing medication with oral anticoagulant drugs. In addition, 25 patients with a diagnosis of chronic PNP were included. Nerve conduction studies and SSEPs were collected in order to characterize PNP patients, and to exclude impairment of peripheral nerve or posterior column conduction in healthy subjects older than 60 years. Conduction studies were performed at our Clinical Neurophysiology Laboratory (Schwarzer Topas EMG System, Natus Europe, Planegg, Germany) according to the clinical standard.

All participants gave their written informed consent for research.

### STDT and TDMT

Somatosensory temporal discrimination threshold was tested with surface electrodes (anode and cathode with 1 mm diameter and 1.5 cm distance in between) placed over the thenar or dorsal foot region. Pairs of square wave electric stimuli with a duration of 0.2 ms were provided by a constant current stimulator (Digitimer, Welwyn Garden City, United Kingdom). Stimulation intensity was determined individually by providing stimuli with stepwise increasing current until participants were able to perceive stimuli clearly (i.e., 10 out of 10 attempts). ISIs were presented in an ascending sequence, starting from 0 ms, in steps of 5 ms. STDT was defined as the shortest ISI when participants perceived two separated pulses in three successive intervals (Tinazzi et al., 1999, 2013a). The mean value of three runs was taken for further analysis.

TDMT was measured following the procedure described by Tinazzi et al. (2005). An insulated tungsten needle microelectrode was inserted at the motor point of the FCR or the TA muscle. The motor point was determined as the cathode position with maximum muscle contraction at stimulation by a surface electrode. The anode was a surface electrode placed 3–4 cm distally to the cathode. Pairs of subsequent electric stimuli (0.2 ms duration, 1–2 mA intensity, below individual stimulus intensity of STDT testing in all cases) with increasing and decreasing ISI (2 runs each) were provided. TDMT was defined as the shortest ISI at which subjects were able to clearly (i.e., three times in a row) identify two separate movements of wrist flexion or foot dorsal extension. A movement consisted of a distinct perceptible muscle contraction without feeling pain or discomfort, along with an observable slight wrist flexion or foot dorsal extension, respectively (Tinazzi et al., 2005). To minimize possible distraction by external stimuli participants wore earplugs and sleep masks.

The mean value of four runs was taken for further analysis.

### Proprioceptive Testing

**FIGURE 1:**
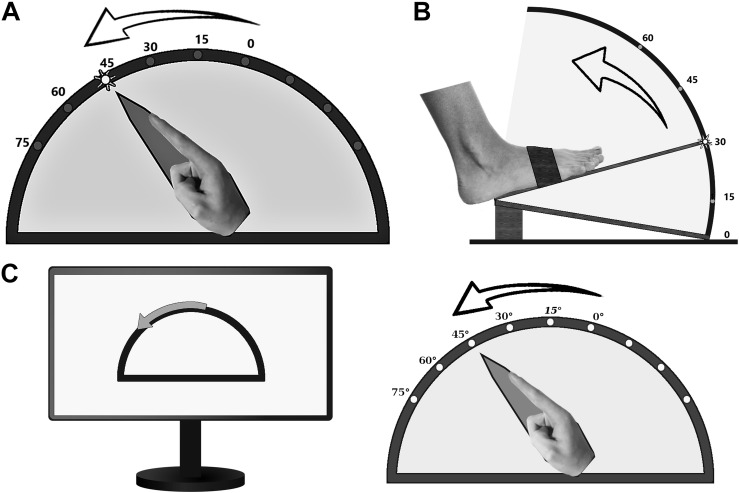
Custom-made goniometers to quantify pointing or estimation errors of **(A)** the upper and **(B)** the lower limb. **(C)** ARROW task: example of a computer screen instruction (left part) and the corresponding active movement of the upper limb.

Proprioception of limbs was assessed by custom-made goniometers without visual feedback of the respective extremity throughout the testing procedure (Figure 1). The device for the upper extremity was built to assess wrist flexion in a range of 0°–75° (Figure 1A), the one for the lower extremity measured foot dorsiflexion in a range of 0°–60° (Figure 1B). Position 0° marks the starting point of motion in our experiment, which corresponds to 15° hand extension and 30° plantar flexion relating to neutral zero method. The goniometers allowed the investigator to monitor the movements directly and to quantify their extent on a scale placed outside the box, invisible for the participant. The respective extremity was fixed with splints and tapes in order to exclude other joint movements.

Subjects were instructed to perform three different proprioceptive tasks as precisely as possible, and to initiate limb movements only on explicit request by the investigator. The first task (LED) comprised pointing to LED markers attached to the outside of the device at 15°, 30°, 45°, 60°, and 75° for the upper limbs (Figure 1A), and 15°, 30°, 45°, and 60° for the right foot (Figure 1B). The participants were instructed to point to the one lighted LED (e.g., 45° in Figure 1A) by one single and quick movement. The examiner documented the reached position, and the subject was asked to return to the starting position. Thereafter, another LED was activated, and the procedure started from its beginning. Each LED lighted up three times during the experiment in a randomized order. In the second task (ARROW), participants were asked to move the respective limb in proportion to curved arrows of different angular dimensions (15°, 30°, or 45°), which were shown on a computer screen (Figure 1C). Thereby, subjects were instructed to imitate the range of movement symbolized by the arrow length. Each length was presented three times in a randomized order. The basic testing procedure was otherwise similar to LED task. In the third task (PASSIVE), which was only performed by the upper limbs, subjects were asked to estimate the position of their limb after passive movements by the experimenter (right side 9°, 21°, 31°, 49°, 63° and left side 13°, 25°, 43°, 51°, 65°, each angle presented twice, one after another). Only for this task, an additional scale was installed at the front of the device so that the participants were able to indicate the felt position of the index finger by telling the corresponding number on the scale. Pointing (LED, ARROW) or estimation (PASSIVE) errors (in degree) were taken as a measure of proprioceptive performance. Conduction of the entire assessment took an average of 30 min.

### Statistical Analyses

SPSS software (IBM) was used for statistical analyses. We tested for normality by using the Shapiro–Wilk test. As data were not normally distributed, we applied the Mann–Whitney *U* test for group comparisons and the Spearman test for correlations. Statistical significance was set at a level of *p* < 0.05. The Benjamini–Hochberg procedure was used to correct for multiple comparisons.

## Results

### Demographic and Clinical Data

A total of 54 healthy subjects (37 females) with a median age of 54 (range 30–76) years were included into this study. In addition, 25 patients (10 females) diagnosed with PNP with a median age of 61 (range 46–79) years were included. Demographic and clinical data of PNP patients and a subgroup of age- and sex-matched healthy controls are summarized in Supplementary Table 1.

### STDT and TDMT: Association With Age and PNP

The results of STDT and TDMT assessment are presented in the upper part of Table 1.

**TABLE 1:** Results of proprioception and temporal discrimination tasks.

**Condition**	**Controls (entire cohort)**		**Controls (age and sex matched)**		**PNP patients**		**Level of significance Mann–Whitney *U* test**
				
	**Mean**	**SD**	**Mean**	**SD**	**Mean**	**SD**	***p*-value**
STDT right hand (ms)	81.4	17.8	81.4	20.4	121.8	34.3	< 0.001^∗^
STDT left hand (ms)^§^	79.6	17.7					
STDT right foot (ms)	109.5	20.3	114.9	20.2	157.8	39.6	0.001^∗^
TDMT right hand (ms)	81.3	16.6	83.3	17.1	126.3	33.4	< 0.001^∗^
TDMT left hand (ms)^§^	82.2	15.3					
TDMT right foot (ms)	102.9	17.0	106.8	17.9	155.2	35.1	< 0.001^∗^
LED right hand (°)	3.2	1.9	3.0	1.8	5.9	2.8	< 0.001^∗^
LED left hand (°)^§^	3.6	2.1					
LED right foot (°)	2.7	1.6	2.6	1.4	6.5	2.5	< 0.001^∗^
ARROW right hand (°)	3.2	2.7	2.9	2.9	5.8	2.7	< 0.001^∗^
ARROW left hand (°)^§^	3.2	1.6					
ARROW right foot (°)	2.6	1.4	2.3	1.2	8.4	4.1	< 0.001^∗^
PASSIVE right hand (°)	2.2	1.1	2.5	1.3	2.2	1.6	0.132
PASSIVE left hand (°)^§^	1.7	1.0					

*^§^ Task not performed in PNP group. ^∗^Significant after Benjamini–Hochberg adjustment. STDT: somatosensory temporal discrimination threshold, TDMT: temporal motor discrimination threshold, PNP: polyneuropathy, SD: standard deviation.*

**FIGURE 2:**
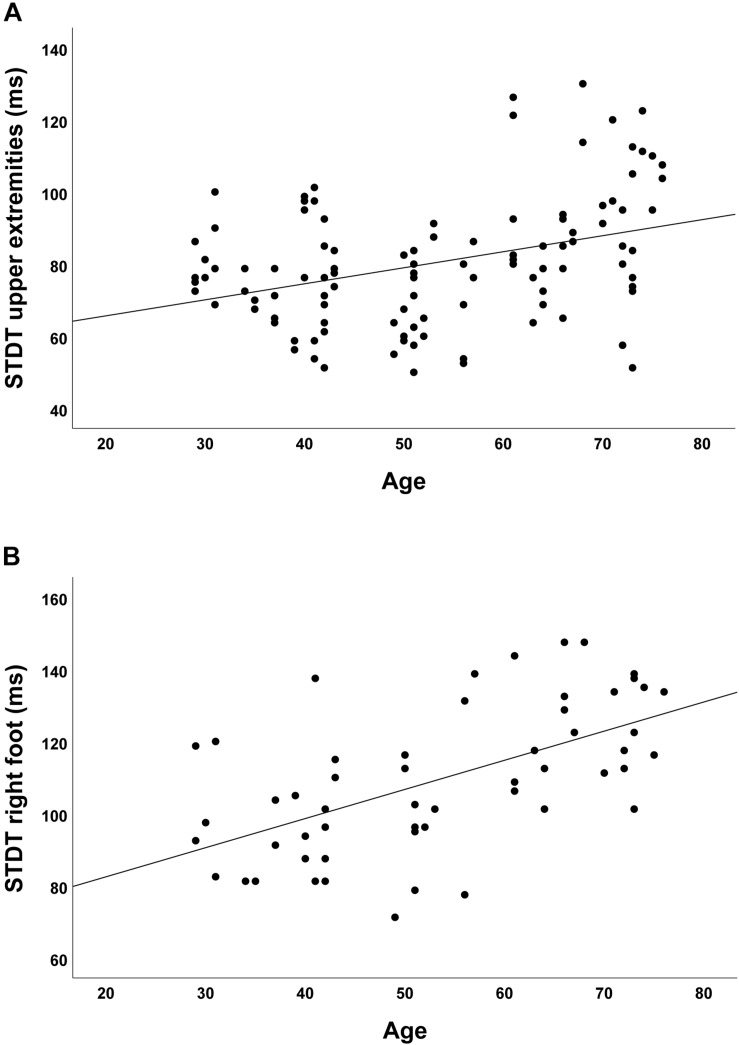
Correlations of STDT of **(A)** upper extremities and **(B)** foot with age in healthy controls.

**FIGURE 3:**
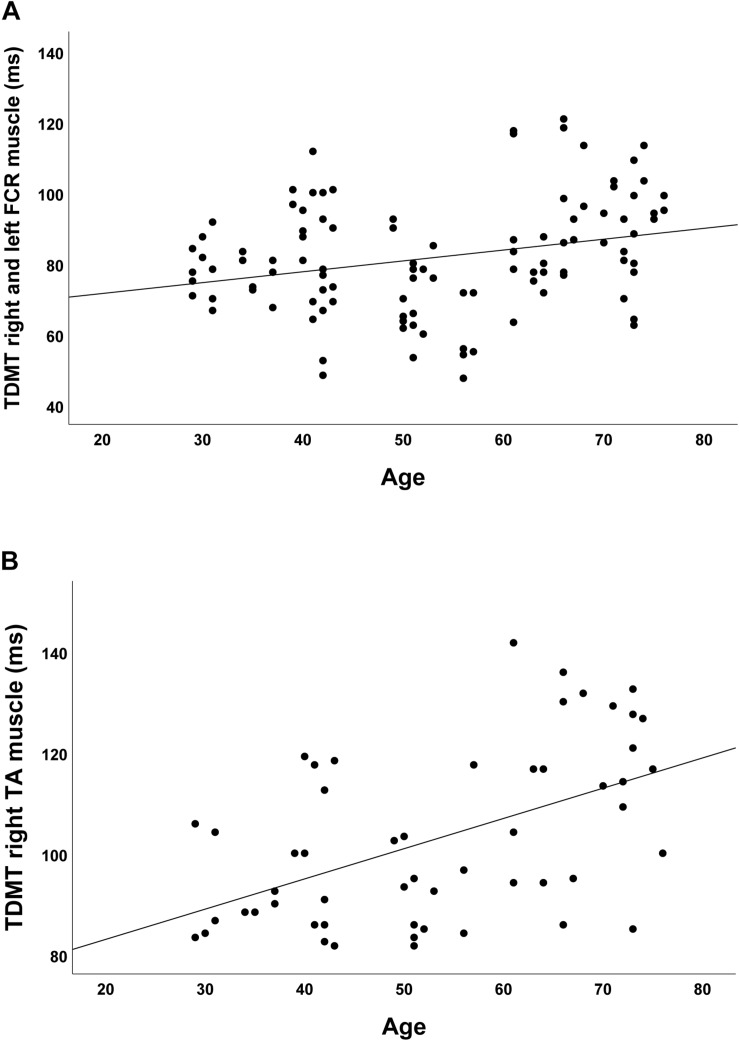
Correlations of TDMT of **(A)** FCR and **(B)** TA muscle with age in healthy controls.

In the group of healthy controls, higher age was associated with higher discrimination threshold levels for STDT of the upper extremities (*r* = 0.348; *p* < 0.001; Figure 2A) and the foot (*r* = 0.581; *p* < 0.001; Figure 2B). Moreover, higher age was associated with higher TDMT levels of the FCR (*r* = 0.267; *p* = 0.005; Figure 3A) and the TA muscle (*r* = 0.465; *p* < 0.001; Figure 3B; all significant after Benjamini–Hochberg adjustment).

Compared to matched controls, patients with PNP showed elevated STDT and TDMT values, with higher thresholds for the upper and lower limbs (Table 1).

### Kinesthesia: Association With Age and PNP

The results of the proprioceptive testing procedures (LED, ARROW, PASSIVE) are presented in the lower part of Table 1.

In the whole group of healthy controls there was no significant correlation between age and higher pointing errors in all performed tasks.

Compared to the group of matched healthy participants, patients with PNP performed worse in the pointing tasks (LED, ARROW), with higher pointing errors for the upper and lower limbs. In the PASSIVE condition, performance was comparable between patients and controls (Table 1).

### STDT, TDMT, and Kinesthesia

**FIGURE 4:**
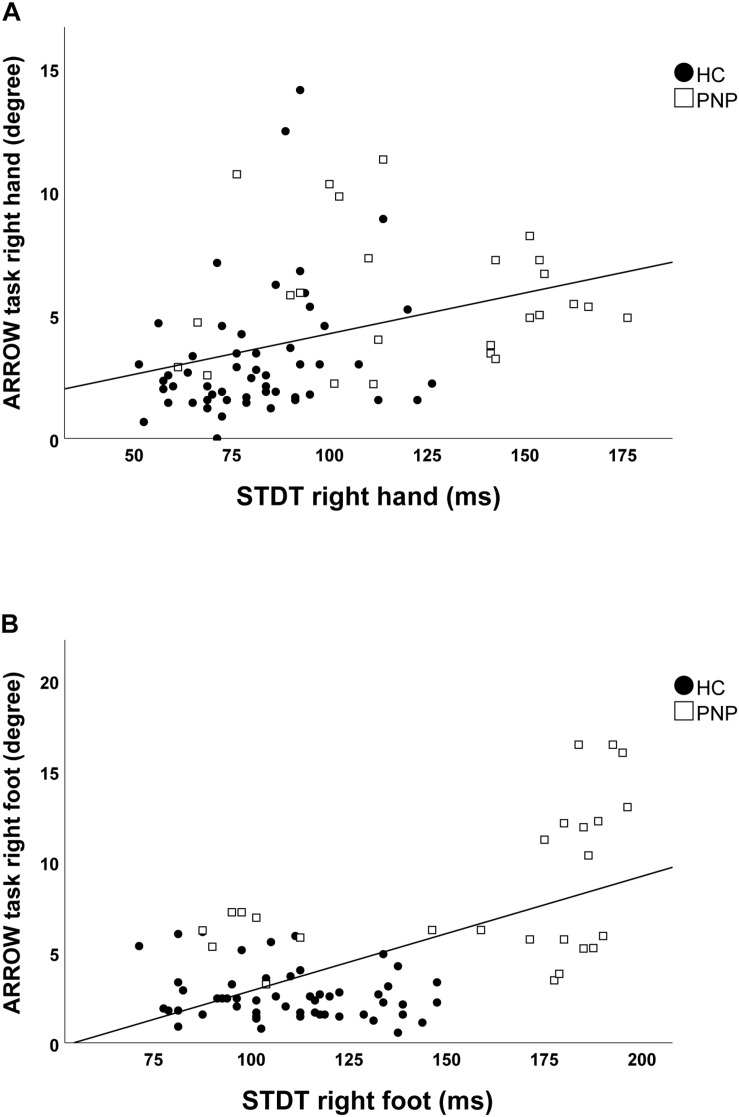
Correlations of STDT of **(A)** hand and **(B)** foot with ARROW task performance in the total cohort (healthy subjects and PNP patients).

**FIGURE 5:**
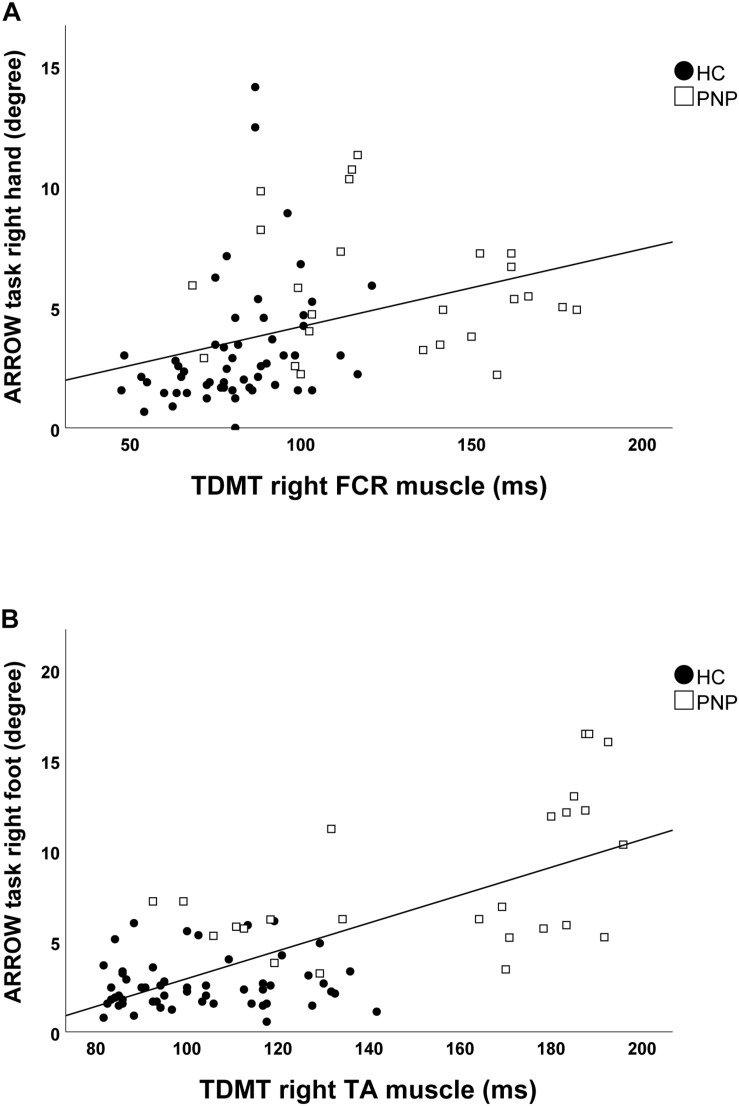
Correlations of TDMT of **(A)** FCR and **(B)** TA muscle with ARROW task performance in the total cohort (healthy subjects and PNP patients).

**FIGURE 6:**
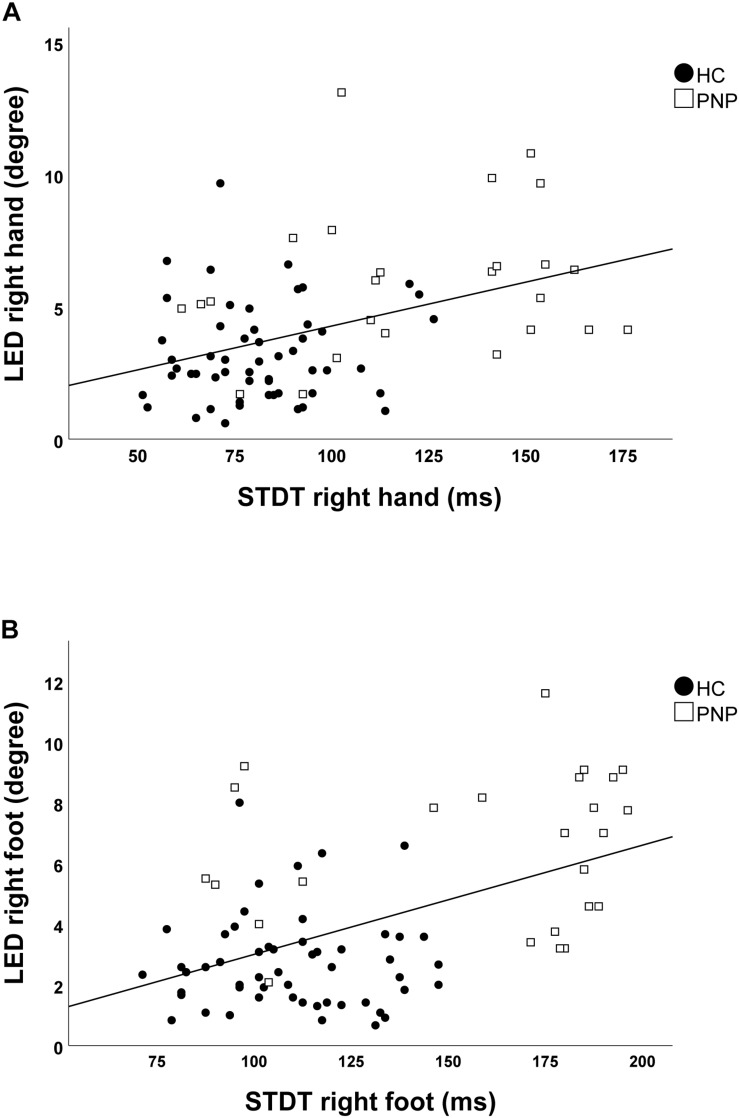
Correlations of STDT of **(A)** hand and **(B)** foot with LED task performance in the total cohort (healthy subjects and PNP patients).

**FIGURE 7:**
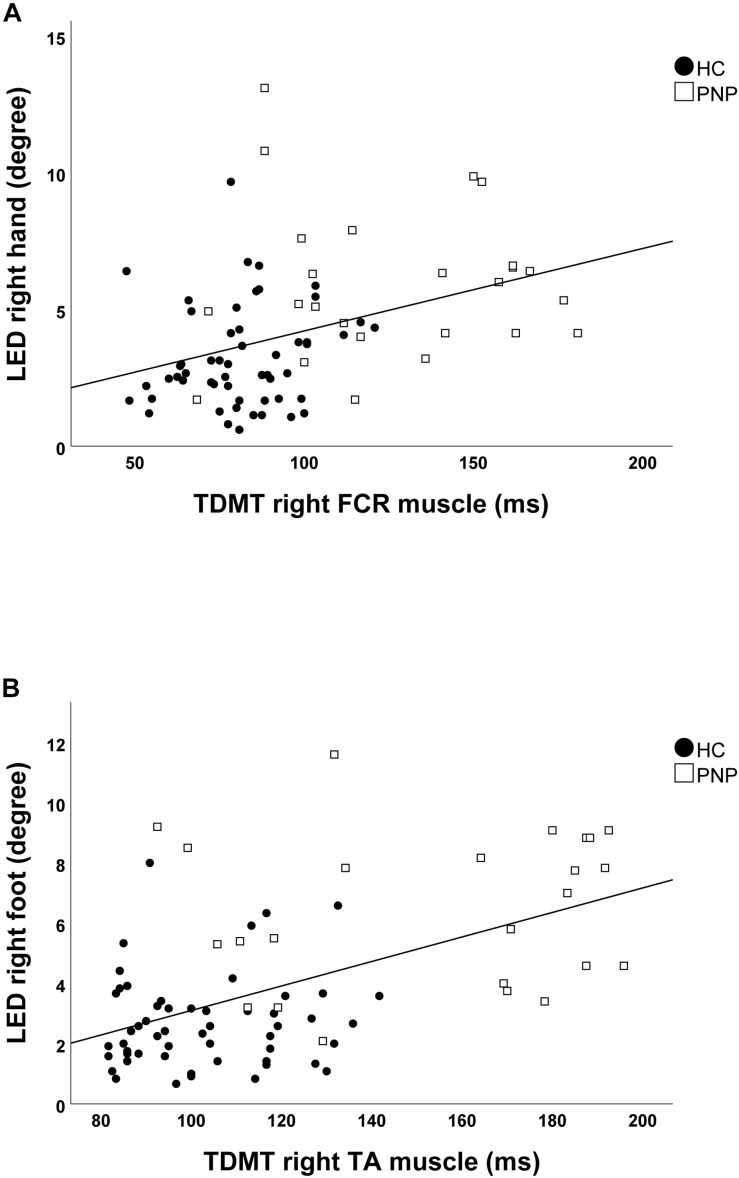
Correlations of TDMT of **(A)** FCR and **(B)** TA muscle with LED task performance in the total cohort (healthy subjects and PNP patients).

Screening the entire data for a potential correlation between the two domains, we found higher temporal discrimination thresholds to be associated with higher pointing errors in the ARROW task at upper (STDT right hand: *r* = 0.477; *p* < 0.001; Figure 4A/TDMT right FCR muscle: *r* = 0.546; *p* < 0.001; Figure 5A) and lower limbs (STDT right foot: *r* = 0.336; *p* = 0.002; Figure 4B/TDMT right TA muscle: *r* = 0.523; *p* < 0.001; Figure 5B). Comparable correlations were found for the LED pointing task: STDT and TDMT correlated significantly with pointing errors (STDT right hand: *r* = 0.369; *p* = 0.001; Figure 6A/TDMT right FCR muscle: *r* = 0.435; *p* < 0.001; Figure 7A/STDT right foot: *r* = 0.372; *p* = 0.001; Figure 6B/TDMT right TA muscle: *r* = 0.460; *p* < 0.001; Figure 7B). All these correlations were significant after Benjamini–Hochberg procedure.

In contrast, there was no significant correlation of temporal discrimination thresholds with performance in the PASSIVE estimation task.

## Discussion

In the present study we assessed temporal discrimination thresholds (STDT and TDMT) and proprioceptive performance in a group of healthy controls and PNP patients. Our main goal was to determine the influence of age and peripheral nerve conduction on both measures as groundwork for their use in patients with movement disorders. In line with our first hypothesis, higher age and PNP were associated with increased discrimination thresholds. Our second hypothesis, in contrast, was only partially confirmed: PNP, but not age, was associated with decreased proprioceptive performance.

Across healthy subjects of different age groups, we found higher age to be correlated with increased temporal discrimination values. In line with a previous study (Ramos et al., 2016), we found positive correlations of age and STDT for the upper limbs, and extended this finding to the lower limbs. Beyond STDT, we found a significant association of higher age with higher TDMT levels of the FCR and an even stronger correlation with TDMT of the TA muscle. Indeed, to the best of our knowledge, this is the first study to demonstrate feasibility of the TDMT paradigm at lower limbs. Together, our data strongly supports the hypothesis that both STDT and TDMT performances decrease during aging. As PNP had been excluded clinically and neurophysiologically in all elderly controls, age-dependent decline in TDT and TDMT may most likely be attributed to changes within central circuits, with several potential reasons (Ramos et al., 2016): Beside a decrease of GABA-ergic neurotransmission in aging (Mora et al., 2008; Lehmann et al., 2012), a reduction of neuronal plasticity within the somatosensory cortex is supposed to play a crucial role in this process (Pellicciari et al., 2009). The latter may be supported by recent findings of an STDT improvement after high frequency repetitive sensory stimulation in healthy subjects which was less prominent in older participants (Erro et al., 2016). The contribution of (subclinical) neurocognitive deficits, which are more frequent in higher age, in temporal perception remains uncertain (Gibbon et al., 1984; Allman and Meck, 2012).

Our finding of significantly increased STDT and TDMT at the upper and lower limbs in patients with PNP points to a considerable impact of peripheral nerve conduction on temporal discrimination. Indeed, blurred signal conduction due to temporal dispersion within the peripheral nerve might well explain our observation of poor temporal discrimination in patients with PNP. As a consequence, peripheral conditions with high general prevalence, like PNP, carpal tunnel syndrome, or radicular compression, might inevitably limit the individual validity of temporal discrimination thresholds in subjects with movement disorders and additional peripheral conditions.

As a potential limitation, the group of PNP patients was rather heterogenous with respect to type and severity of neuropathy. While an increase of STDT and TDMT in demyelinating PNP might be more suggestive than in axonal PNP or small fiber neuropathy, the respective impact of different types of nerve damage remains speculative. Moreover, nerve conduction studies predominantly reveal information about the distal part of the nerve, whereas proximal demyelination may be missed easily. Consequently, the attempt to disentangle the causal relationship between a particular finding in nerve conduction studies and temporal discrimination performance would be an overinterpretation of our data. This is even more so since the CNS would not remain unaffected by peripheral neuropathy – plastic processes like cortical rearrangement following PNP-related partial denervation might also affect temporal discrimination on the CNS level.

Notably, we did not find a *differential* association of STDT and TDMT with age or PNP: both parameters changed concordantly, i.e., they were positively correlated with age, and both were similarly increased in PNP patients as compared to healthy subjects. Thus, physiological aging as well as PNP influence STDT and TDMT similarly. This is in line with the observation that specific patterns of altered temporal discrimination in disorders like essential tremor or dystonia can be attributed to specific changes at the level of the CNS. Several brain areas have been suggested by fMRI to be involved in temporal discrimination, first of all the basal ganglia (Pastor et al., 2004), in particular the putamen (Kimmich et al., 2014), and the superior colliculus (Hutchinson et al., 2014; Mc Govern et al., 2017). In addition, involvement of the prefrontal cortex, anterior cingulate, pre-supplementary motor area, precentral gyrus, sensorimotor cortex, inferior parietal lobule, and cerebellum has been observed (Pastor et al., 2004; Di Biasio et al., 2015; Rocchi et al., 2017; Erro et al., 2018).

We did not find a significant correlation between age and pointing or estimation errors, respectively. Earlier studies have demonstrated an age-related decline of passive finger (Ko et al., 2015; Zhang et al., 2015; Ingemanson et al., 2016; Rinderknecht et al., 2017) and ankle (Ko et al., 2015) proprioception, while age had no effect on the accuracy rate in a pointing task under restricted visual feedback conditions (Zhang et al., 2015). In the light of the different aspects of proprioception assessed at different joints in these studies, our findings would be compatible with an age-dependent decline in passive, but not active proprioceptive performance, which might be restricted to distal joints and therefore, not appear at the wrist. In general, it is still under debate whether passive and active proprioception are different neuronal concepts and potentially involve different central circuits (Gritsenko et al., 2007; Capaday et al., 2013). In addition, we cannot exclude the possibility that our PASSIVE task at the wrist might have lacked the precision to detect very small angular differences, for example due to the fact that the movement was performed by the experimenter rather than an electric device or to limited control for cues from the skin touching the goniometer splint.

Patients with PNP showed an impaired kinesthetic performance as evidenced by higher pointing errors compared to controls. In the light of reduced peripheral afferent input due to PNP, this finding is not surprising (Rothwell et al., 1982; Sainburg et al., 1995). As for the PASSIVE task, we consider it likely that the low sensitivity of an assessment at the wrist (compared to finger joints) in addition to the methodological limitations discussed above might explain the lack of significant differences between PNP patients and matched controls. As our foot goniometer did not allow movements with gravity eliminated, the PASSIVE condition was only performed at the upper extremities, where, from clinical experience, proprioceptive dysfunction due to PNP is less prominent.

As an additional observation, we found correlations between STDT and TDMT and the performance in two different active pointing tasks: Higher temporal discrimination thresholds were associated with higher pointing errors in the ARROW and LED task in upper and lower extremities. Though postulated by earlier studies, which had found differential alterations of STDT and TDMT in patients with movement disorders (Artieda et al., 1992; Bara-Jimenez et al., 2000; Tinazzi et al., 2006; Fiorio et al., 2007), the association of both parameters with behavioral measures of proprioception had never been systematically assessed.

Active pointing movements rely on permanent “on-line” adjustments of their temporospatial properties in order to achieve a high level of precision (Georgopoulos, 2002). Temporal discrimination is supposed to involve a complex network process within the CNS subserving conscious evaluation of double stimuli, i.e., to answer the question whether a subject feels a single or a double stimulus. It therefore, seems worth speculating on whether temporal discrimination thresholds and pointing precision may be considered different measures of one and the same network. In this case, their correlation would support the notion that kinesthesia is indeed the link between impaired temporal discrimination and neurological conditions in several movement disorders (Tinazzi et al., 2005, 2013b; Fiorio et al., 2007).

As correlations do not allow directional or causal inference, an alternative explanation might be that both temporal discrimination and pointing performance are modulated comparably by a common third parameter. This could either be an unidentified parameter or simply peripheral nerve conduction with PNP-related variability. In the latter case we would expect a lack of correlation when PNP patients are excluded. However, there remained to be a significant correlation between STDT and TDMT and ARROW task in upper limbs in the group of healthy subjects, which supports an association of the two parameters above their common modulation by peripheral conduction.

Functional imaging studies on proprioception during either vibration induced illusory motion or passive extremity movements revealed involvement of a number of brain regions partially overlapping with those discussed in the context of temporal discrimination (Weiller et al., 1996; Gelnar et al., 1998; Mima et al., 1999; Francis et al., 2000; Romaiguere et al., 2003; Naito et al., 2007; Kavounoudias et al., 2008). It is beyond the scope of our study to speculate about the neural underpinnings of both phenomena in the CNS. However, future research should specifically assess proprioceptive performance by means of a “top-down based” task in the particular movement disorders to probe for behavioral correlates of differential STDT and TDMT changes. Functional imaging and non-invasive brain stimulation might complement such studies in order to identify brain structures involved in both processes.

## Conclusion

Age and PNP have significant impact on measures of temporal discrimination and/or proprioceptive capacity. STDT and TDMT increase with age, and PNP is associated with higher STDT/TDMT values and reduced precision in pointing tasks. If applied in studies on movement disorder, where STDT/TDMT may be used in order to define corresponding endophenotypes, it is important to account for these factors to increase validity of the measurements. This is particularly important in view of a significantly higher prevalence of PNP in patients with PD as compared to controls (Conradt et al., 2018). As an additional observation of high interest, higher error rates in pointing tasks correlate with elevated discrimination thresholds. This may prompt further studies on the presumptive link between these two domains and their potential use as endophenotypic markers in neurological conditions.

## Data Availability Statement

All datasets generated for this study are included in the article/Supplementary Material.

## Ethics Statement

The studies involving human participants were reviewed and approved by Ethik-Kommission der Universität Würzburg. The patients/participants provided their written informed consent to participate in this study.

## Author Contributions

TO and DZ designed the study concept and wrote the manuscript with the help of TW. TW and TO carried out the experiments and performed the statistical data analysis.

## Conflict of Interest

The authors declare that the research was conducted in the absence of any commercial or financial relationships that could be construed as a potential conflict of interest.

## Abbreviations

CNS: central nervous system
EMG: electromyography
FCR: flexor carpi radialis muscle
fMRI: functional magnetic resonance imaging
GABA: gamma-aminobutyric acid
ISI: interstimulus interval
LED: light-emitting diode
PD: Parkinson’s disease
PNP: polyneuropathy
SSEP: somatosensory evoked potential
STDT: somatosensory temporal discrimination threshold
TA: tibialis anterior muscle
TDMT: temporal discrimination movement threshold.

## References

[B1] Aglioti S. M. Fiorio M. Forster B. Tinazzi M. (2003). Temporal discrimination of cross-modal and unimodal stimuli in generalized dystonia. *Neurology* 60 782–785. 10.1212/01.wnl.0000046528.24693.5b 12629233

[B2] Allman M. J. Meck W. H. (2012). Pathophysiological distortions in time perception and timed performance. *Brain* 135 656–677. 10.1093/brain/awr210 21921020 PMC3491636

[B3] Artieda J. Pastor M. A. Lacruz F. Obeso J. A. (1992). Temporal discrimination is abnormal in Parkinson’s disease. *Brain* 115(Pt 1), 199–210. 10.1093/brain/115.1.199 1559154

[B4] Bara-Jimenez W. Shelton P. Sanger T. D. Hallett M. (2000). Sensory discrimination capabilities in patients with focal hand dystonia. *Ann. Neurol.* 47 377–380. 10.1002/1531-8249(200003)47:3<377::aid-ana16>3.3.co;2-u 10716260

[B5] Capaday C. Darling W. G. Stanek K. Van Vreeswijk C. (2013). Pointing to oneself: active versus passive proprioception revisited and implications for internal models of motor system function. *Exp. Brain Res.* 229 171–180. 10.1007/s00221-013-3603-4 23756602

[B6] Conradt C. Guo D. Miclea A. Nisslein T. Ismail C. Chatamra K. (2018). Increased prevalence of polyneuropathy in parkinson’s disease patients: an observational study. *J. Parkinsons Dis.* 8 141–144. 10.3233/jpd-161057 29154292 PMC5836410

[B7] Conte A. Leodori G. Ferrazzano G. De Bartolo M. I. Manzo N. Fabbrini G. (2016). Somatosensory temporal discrimination threshold in Parkinson’s disease parallels disease severity and duration. *Clin. Neurophysiol.* 127 2985–2989. 10.1016/j.clinph.2016.06.026 27458837

[B8] Conte A. Modugno N. Lena F. Dispenza S. Gandolfi B. Iezzi E. (2010). Subthalamic nucleus stimulation and somatosensory temporal discrimination in Parkinson’s disease. *Brain* 133 2656–2663. 10.1093/brain/awq191 20802206

[B9] Di Biasio F. Conte A. Bologna M. Iezzi E. Rocchi L. Modugno N. (2015). Does the cerebellum intervene in the abnormal somatosensory temporal discrimination in Parkinson’s disease? *Parkinsonism Relat. Disord.* 21 789–792. 10.1016/j.parkreldis.2015.04.004 25922270

[B10] Erro R. Rocchi L. Antelmi E. Liguori R. Tinazzi M. Berardelli A. (2018). High frequency somatosensory stimulation in dystonia: evidence fordefective inhibitory plasticity. *Mov. Disord.* 33 1902–1909. 10.1002/mds.27470 30376603

[B11] Erro R. Rocchi L. Antelmi E. Palladino R. Tinazzi M. Rothwell J. (2016). High frequency repetitive sensory stimulation improves temporal discrimination in healthy subjects. *Clin. Neurophysiol.* 127 817–820. 10.1016/j.clinph.2015.06.023 26149631

[B12] Fiorio M. Stanzani C. Rothwell J. C. Bhatia K. P. Moretto G. Fiaschi A. (2007). Defective temporal discrimination of passive movements in Parkinson’s disease. *Neurosci. Lett.* 417 312–315. 10.1016/j.neulet.2007.02.050 17367930

[B13] Fiorio M. Tinazzi M. Bertolasi L. Aglioti S. M. (2003). Temporal processing of visuotactile and tactile stimuli in writer’s cramp. *Ann. Neurol.* 53 630–635. 10.1002/ana.10525 12730997

[B14] Fiorio M. Tinazzi M. Scontrini A. Stanzani C. Gambarin M. Fiaschi A. (2008). Tactile temporal discrimination in patients with blepharospasm. *J. Neurol. Neurosurg. Psychiatry* 79 796–798. 10.1136/jnnp.2007.131524 17986501

[B15] Francis S. T. Kelly E. F. Bowtell R. Dunseath W. J. Folger S. E. Mcglone F. (2000). fMRI of the responses to vibratory stimulation of digit tips. *Neuroimage* 11 188–202. 10.1006/nimg.2000.0541 10694461

[B16] Gelnar P. A. Krauss B. R. Szeverenyi N. M. Apkarian A. V. (1998). Fingertip representation in the human somatosensory cortex: an fMRI study. *Neuroimage* 7 261–283. 10.1006/nimg.1998.0341 9626668

[B17] Georgopoulos A. P. (2002). Cognitive motor control: spatial and temporal aspects. *Curr. Opin. Neurobiol.* 12 678–683. 10.1016/s0959-4388(02)00382-3 12490258

[B18] Gibbon J. Church R. M. Meck W. H. (1984). Scalar timing in memory. *Ann. N. Y. Acad. Sci.* 423 52–77. 10.1111/j.1749-6632.1984.tb23417.x 6588812

[B19] Gritsenko V. Krouchev N. I. Kalaska J. F. (2007). Afferent input, efference copy, signal noise, and biases in perception of joint angle during active versus passive elbow movements. *J. Neurophysiol.* 98 1140–1154. 10.1152/jn.00162.2007 17615137

[B20] Hutchinson M. Isa T. Molloy A. Kimmich O. Williams L. Molloy F. (2014). Cervical dystonia: a disorder of the midbrain network for covert attentional orienting. *Front. Neurol.* 5:54. 10.3389/fneur.2014.00054 24803911 PMC4009446

[B21] Ingemanson M. L. Rowe J. B. Chan V. Wolbrecht E. T. Cramer S. C. Reinkensmeyer D. J. (2016). Use of a robotic device to measure age-related decline in finger proprioception. *Exp. Brain Res.* 234 83–93. 10.1007/s00221-015-4440-4 26378004 PMC9153390

[B22] Kavounoudias A. Roll J. P. Anton J. L. Nazarian B. Roth M. Roll R. (2008). Proprio-tactile integration for kinesthetic perception: an fMRI study. *Neuropsychologia* 46 567–575. 10.1016/j.neuropsychologia.2007.10.002 18023825

[B23] Kimmich O. Molloy A. Whelan R. Williams L. Bradley D. Balsters J. (2014). Temporal discrimination, a cervical dystonia endophenotype: penetrance and functional correlates. *Mov. Disord.* 29 804–811. 10.1002/mds.25822 24482092

[B24] Ko S. U. Simonsick E. Deshpande N. Ferrucci L. (2015). Sex-specific age associations of ankle proprioception test performance in older adults: results from the Baltimore Longitudinal Study of Aging. *Age Ageing* 44 485–490. 10.1093/ageing/afv005 25637144 PMC4411223

[B25] Lee M. J. Son J. S. Lee J. H. Kim S. J. Lyoo C. H. Lee M. S. (2016). Impact of prolonged temporal discrimination threshold on finger movements of Parkinson’s disease. *PLoS One* 11:e0167034. 10.1371/journal.pone.0167034 27893840 PMC5125668

[B26] Lehmann K. Steinecke A. Bolz J. (2012). GABA through the ages: regulation of cortical function and plasticity by inhibitory interneurons. *Neural Plast.* 2012:892784. 10.1155/2012/892784 22792496 PMC3390141

[B27] Mc Govern E. M. Killian O. Narasimham S. Quinlivan B. Butler J. B. Beck R. (2017). Disrupted superior collicular activity may reveal cervical dystonia disease pathomechanisms. *Sci. Rep.* 7:16753. 10.1038/s41598-017-17074-x 29196716 PMC5711841

[B28] Mima T. Sadato N. Yazawa S. Hanakawa T. Fukuyama H. Yonekura Y. (1999). Brain structures related to active and passive finger movements in man. *Brain* 122(Pt 10), 1989–1997. 10.1093/brain/122.10.1989 10506099

[B29] Mora F. Segovia G. Del Arco A. (2008). Glutamate-dopamine-GABA interactions in the aging basal ganglia. *Brain Res. Rev.* 58 340–353. 10.1016/j.brainresrev.2007.10.006 18036669

[B30] Naito E. Nakashima T. Kito T. Aramaki Y. Okada T. Sadato N. (2007). Human limb-specific and non-limb-specific brain representations during kinesthetic illusory movements of the upper and lower extremities. *Eur. J. Neurosci.* 25 3476–3487. 10.1111/j.1460-9568.2007.05587.x 17553017

[B31] Pastor M. A. Day B. L. Macaluso E. Friston K. J. Frackowiak R. S. (2004). The functional neuroanatomy of temporal discrimination. *J. Neurosci.* 24 2585–2591. 10.1523/jneurosci.4210-03.2004 15014134 PMC6729480

[B32] Pellicciari M. C. Miniussi C. Rossini P. M. De Gennaro L. (2009). Increased cortical plasticity in the elderly: changes in the somatosensory cortex after paired associative stimulation. *Neuroscience* 163 266–276. 10.1016/j.neuroscience.2009.06.013 19524024

[B33] Ramos V. F. Esquenazi A. Villegas M. A. Wu T. Hallett M. (2016). Temporal discrimination threshold with healthy aging. *Neurobiol. Aging* 43 174–179. 10.1016/j.neurobiolaging.2016.04.009 27255827 PMC4893195

[B34] Riemann B. L. Lephart S. M. (2002a). The sensorimotor system, part I: the physiologic basis of functional joint stability. *J. Athl. Train* 37 71–79. 16558670 PMC164311

[B35] Riemann B. L. Lephart S. M. (2002b). The sensorimotor system, Part II: the role of proprioception in motor control and functional joint stability. *J. Athl. Train* 37 80–84. 16558671 PMC164312

[B36] Rinderknecht M. D. Lambercy O. Raible V. Liepert J. Gassert R. (2017). Age-based model for metacarpophalangeal joint proprioception in elderly. *Clin. Interv. Aging* 12 635–643. 10.2147/CIA.S129601 28435235 PMC5388205

[B37] Rocchi L. Conte A. Nardella A. Li Voti P. Di Biasio F. Leodori G. (2013). Somatosensory temporal discrimination threshold may help to differentiate patients with multiple system atrophy from patients with Parkinson’s disease. *Eur. J. Neurol.* 20 714–719. 10.1111/ene.12059 23278905

[B38] Rocchi L. Erro R. Antelmi E. Berardelli A. Tinazzi M. Liguori R. (2017). High frequency somatosensory stimulation increases sensori-motor inhibition and leads to perceptual improvement in healthy subjects. *Clin. Neurophysiol.* 128 1015–1025. 10.1016/j.clinph.2017.03.046 28463818

[B39] Romaiguere P. Anton J. L. Roth M. Casini L. Roll J. P. (2003). Motor and parietal cortical areas both underlie kinaesthesia. *Brain Res. Cogn. Brain Res.* 16 74–82. 10.1016/s0926-6410(02)00221-5 12589891

[B40] Rothwell J. C. Traub M. M. Day B. L. Obeso J. A. Thomas P. K. Marsden C. D. (1982). Manual motor performance in a deafferented man. *Brain* 105(Pt 3), 515–542. 10.1093/brain/105.3.515 6286035

[B41] Sainburg R. L. Ghilardi M. F. Poizner H. Ghez C. (1995). Control of limb dynamics in normal subjects and patients without proprioception. *J. Neurophysiol.* 73 820–835. 10.1152/jn.1995.73.2.820 7760137 PMC11102602

[B42] Tinazzi M. Fasano A. Di Matteo A. Conte A. Bove F. Bovi T. (2013a). Temporal discrimination in patients with dystonia and tremor and patients with essential tremor. *Neurology* 80 76–84. 10.1212/WNL.0b013e31827b1a54 23243072

[B43] Tinazzi M. Morgante F. Peretti A. Mariotti C. Panzeri M. Fiorio M. (2013b). Impaired temporal processing of tactile and proprioceptive stimuli in cerebellar degeneration. *PLoS One* 8:e78628. 10.1371/journal.pone.0078628 24244328 PMC3823840

[B44] Tinazzi M. Fasano A. Peretti A. Bove F. Conte A. Dallocchio C. (2014). Tactile and proprioceptive temporal discrimination are impaired in functional tremor. *PLoS One* 9:e102328. 10.1371/journal.pone.0102328 25051180 PMC4106827

[B45] Tinazzi M. Fiorio M. Bertolasi L. Aglioti S. M. (2004). Timing of tactile and visuo-tactile events is impaired in patients with cervical dystonia. *J. Neurol.* 251 85–90. 10.1007/s00415-004-0282-x 14999494

[B46] Tinazzi M. Fiorio M. Stanzani C. Moretto G. Smania N. Fiaschi A. (2006). Temporal discrimination of two passive movements in writer’s cramp. *Mov. Disord.* 21 1131–1135. 10.1002/mds.20892 16628603

[B47] Tinazzi M. Frasson E. Bertolasi L. Fiaschi A. Aglioti S. (1999). Temporal discrimination of somesthetic stimuli is impaired in dystonic patients. *Neuroreport* 10 1547–1550. 10.1097/00001756-199905140-00028 10380978

[B48] Tinazzi M. Stanzani C. Fiorio M. Smania N. Moretto G. Fiaschi A. (2005). Temporal discrimination of two passive movements in humans: a new psychophysical approach to assessing kinaesthesia. *Exp. Brain Res.* 166 184–189. 10.1007/s00221-005-2353-3 16021430

[B49] Weiller C. Juptner M. Fellows S. Rijntjes M. Leonhardt G. Kiebel S. (1996). Brain representation of active and passive movements. *Neuroimage* 4 105–110. 10.1006/nimg.1996.0034 9345502

[B50] Zhang L. Yang J. Inai Y. Huang Q. Wu J. (2015). Effects of aging on pointing movements under restricted visual feedback conditions. *Hum. Mov. Sci.* 40 1–13. 10.1016/j.humov.2014.11.009 25506638

