# Supplementary material for: Temporal Discrimination Thresholds and Proprioceptive Performance: Impact of Age and Nerve Conduction
Source: Front Neurosci. 2019 Nov 19;13:1241. doi: 10.3389/fnins.2019.01241 (PMC6877661; doi:10.3389/fnins.2019.01241)
Supplement: Supplementary file 1 [file Table_1.DOCX]

| **Pat. No.** | **age range** | **CMAP (mV)** | **mNCV (m/s)** | **SNAP (µV)** | **sNCV (m/s)** | **Diagnosis/Etiology** | **Ctrl. No.** | **age range** |
| --- | --- | --- | --- | --- | --- | --- | --- | --- |
| **1** | 51-55 | 10.7 | 41.9 | 18 | 41 | small fiber neuropathy | **22** | 51-55 |
| **2** | 71-75 | 13.3 | 42.2 | 4.7 | 43.7 | unknown/idiopathic | **50** | 71-75 |
| **3** | 51-55 | 3.6 | 44.3 | 13.6 | 42.4 | unknown/idiopathic | **44** | 56-60 |
| **4** | 46-50 | 7.5 | 39.2 | NR | NR | unknown/idiopathic | **6** | 46-50 |
| **5** | 71-75 | 0.4 | 30.8 | NR | NR | vitamin B12 deficiency | **52** | 71-75 |
| **6** | 66-70 | 0.4 | 35.2 | NR | NR | unknown/idiopathic | **10** | 66-70 |
| **7** | 51-55 | 1.5 | 36.5 | 1.4 | 40.1 | vitamin B12 deficiency | **12** | 51-55 |
| **8** | 51-55 | 1.1 | 26 | 3.3 | 34.2 | unknown/idiopathic | **30** | 51-55 |
| **9** | 76-80 | 8.2 | 34.2 | NR | NR | vitamin B12 deficiency | **48** | 71-75 |
| **10** | 61-65 | 1.0 | 36.7 | NR | NR | vasculitic neuropathy | **32** | 66-70 |
| **11** | 71-75 | 0.2 | 28.6 | NR | NR | unknown/idiopathic | **21** | 71-75 |
| **12** | 56-60 | 17.4 | 40 | 10.3 | 38.7 | unknown/idiopathic | **34** | 61-65 |
| **13** | 51-55 | 9.6 | 32.6 | 10.6 | 46 | CIDP | **29** | 46-50 |
| **14** | 71-75 | 4.7 | 38.3 | 3.5 | 39 | unknown/idiopathic | **53** | 71-75 |
| **15** | 71-75 | NR | NR | NR | NR | unknown/idiopathic | **42** | 71-75 |
| **16** | 61-65 | 0.1 | 33.3 | NR | NR | unknown/idiopathic | **43** | 71-75 |
| **17** | 71-75 | 1.5 | 37.5 | 3.3 | 41 | rheumatoid arthritis | **47** | 71-75 |
| **18** | 51-55 | 1.4 | 48.8 | NR | NR | unknown/idiopathic | **25** | 51-55 |
| **19** | 55-60 | 0.1 | 24.6 | NR | NR | paraproteinaemic PNP | **18** | 61-65 |
| **20** | 71-75 | 3.4 | 37.1 | NR | NR | unknown/idiopathic | **27** | 71-75 |
| **21** | 61-65 | 0.3 | 30.7 | NR | NR | CIDP | **9** | 66-70 |
| **22** | 46-50 | 16.0 | 37.7 | NR | NR | CIDP | **13** | 41-45 |
| **23** | 51-55 | 23.9 | 36.4 | 12.4 | 35.7 | MADSAM | **37** | 51-55 |
| **24** | 61-65 | 0.3 | 34.2 | 2.1 | 37.5 | unknown/idiopathic | **31** | 61-65 |
| **25** | 56-60 | NR | NR | 2.0 | 33.7 | paraproteinaemic PNP | **36** | 56-60 |

(mean age 61.9 ± 9.7, 10 f) (mean age 62.3 ±10.3, 12 f)
