# Supplementary material for: Temporal Discrimination Thresholds and Proprioceptive Performance: Impact of Age and Nerve Conduction
Source: Front Neurosci. 2019 Nov 19;13:1241. doi: 10.3389/fnins.2019.01241 (PMC6877661; doi:10.3389/fnins.2019.01241)
Supplement: Supplementary file 2 [file Data_Sheet_1.docx]

**SUPPLEMENTARY MATERIAL**

***Methods descriptions:***

Conduction studies:

Motor neurography of the tibial nerve was performed by measuring the compound muscle action potential (CMAP) amplitude via surface electrodes on the abductor hallucis muscle after supramaximal electrical stimulation of the nerve at a distal (posterior to medial malleolus) and proximal (popliteal fossa) area. Nerve conduction velocity (NCV) for the lower leg was calculated from the distance and the motor latency differences between proximal and distal stimulations. Sensory neurography of the sural nerve was examined in antidromic stimulation technique. Surface electrodes were positioned posterior to the lateral malleolus, and the nerve was stimulated supramaximal at the lower calf area. The amplitude of the sensory nerve action potential (SNAP; average of 10) and sensory NCV were determined. CMAP and SNAP were measured “peak to peak”. Conduction studies were only performed at a skin temperature of at least 30 °C.

SSEP (average of 200 responses) were registered by needle electrodes placed over the somatosensory cortex (for median nerve SSEP at C3’ and C4’, for tibial nerve SSEP at Cz, according to the international 10-20-system; reference at Fz in both cases) after nerve stimulation at the wrist or behind the medial malleolus, respectively. The mean peak-to-peak amplitudes and N20 or P40 latencies, respectively, were analyzed.

Controls with any abnormality of the neurographies or SSEPs were excluded from the study.
